# OsARF16 Is Involved in Cytokinin-Mediated Inhibition of Phosphate Transport and Phosphate Signaling in Rice (*Oryza sativa L.*)

**DOI:** 10.1371/journal.pone.0112906

**Published:** 2014-11-11

**Authors:** Chenjia Shen, Runqing Yue, Yanjun Yang, Lei Zhang, Tao Sun, Shuanggui Tie, Huizhong Wang

**Affiliations:** 1 College of Life and Environmental Sciences, Hangzhou Normal University, Hangzhou 310036, China; 2 Henan Academy of Agricultural Sciences, Zhengzhou 450002, China; 3 Department of Plant Pathology, Washington State University, Pullman, WA 99164-6430, United States of America; National Taiwan University, Taiwan

## Abstract

**Background:**

Plant responses to phytohormone stimuli are the most important biological features for plants to survive in a complex environment. Cytokinin regulates growth and nutrient homeostasis, such as the phosphate (Pi) starvation response and Pi uptake in plants. However, the mechanisms underlying how cytokinin participates in Pi uptake and Pi signaling are largely unknown. In this study, we found that OsARF16 is required for the cytokinin response and is involved in the negative regulation of Pi uptake and Pi signaling by cytokinin.

**Principal Findings:**

The mutant *osarf16* showed an obvious resistance to exogenous cytokinin treatment and the expression level of the *OsARF16* gene was considerably up-regulated by cytokinin. Cytokinin (6-BA) application suppressed Pi uptake and the Pi starvation response in wild-type *Nipponbare* (NIP) and all these responses were compromised in the *osarf16* mutant. Our data showed that cytokinin inhibits the transport of Pi from the roots to the shoots and that OsARF16 is involved in this process. The Pi content in the *osarf16* mutant was much higher than in NIP under 6-BA treatment. The expressions of *PHOSPHATE TRANSPORTER1* (*PHT1*) genes, *phosphate (Pi) starvation-induced* (*PSI*) genes and *purple PAPase* genes were higher in the *osarf16* mutant than in NIP under cytokinin treatment.

**Conclusion:**

Our results revealed a new biological function for OsARF16 in the cytokinin-mediated inhibition of Pi uptake and Pi signaling in rice.

## Introduction

The phytohormone, auxin, governs many aspects of growth, development and nutrient homeostasis in plants. Auxin perception and signal transduction regulate the expressions of many downstream genes via the AUXIN (AUX)/INDOLE-3ACETIC ACID (IAA) and AUXIN RESPONSE FACTOR (ARF)-mediated auxin signaling pathways [1]. ARFs, which regulate the expression of auxin-responsive genes, are involved in all the developmental processes from embryogenesis to senescence [2]. Many plant physiological functions depend on ARFs, including apical hook formation in seedlings [3], abaxial identity in the gynoecium [4], embryo development and vascular formation [5], hypocotyl tropisms [6], fruit development from fertilization [7], lateral root formation [8] and root system growth [9].

Auxin interacts with various phytohormones to regulate many physiological functions, either synergistically or antagonistically [10]–[13]. Among them, cytokinin is known to act antagonistically to auxin and plays an essential role in controlling plant morphogenesis [14]. Two primary cytokinin response transcription factors, *ARR1* and *ARR12*, activate the *SHORT HYPOCOTYL2* (*SHY2*) gene, which encodes a repressor that regulates the transcription of *PIN-FORMED* (*PIN*) auxin transporter genes [15]. Cytokinin inhibits the expression of auxin transport genes and prevents the establishment of an auxin gradient during lateral root initiation [16]–[18]. *WOX11*, a WUSCHEL-related homeobox gene, is induced by both auxin and cytokinin and represses a cytokinin-signaling negatively regulated gene, *OsRR2* [19]. The mRNA accumulation of *AtARF19*, an auxin response factor involved in cell differentiation, has been shown to rely on the cytokinin-dependent transcription factor, AtARR12 [20]. Embryogenesis and post-embryonic root organ development all depend on the antagonistic interaction between cytokinin and auxin [21].

It has been shown that cytokinin is involved in the phosphate starvation response and phosphate uptake. The expressions of the phosphate transport gene, *AtPT1*, and the *AtPHO1* family genes are suppressed by both auxin and cytokinin [22], [23]. Cytokinin also regulates many Pi deprivation-induced genes. For example, *At4*, which is expressed in the vascular tissue and is responsible for Pi accumulation in the shoots, is regulated by cytokinin [24]. *AtIPS1* and other Pi starvation responsive genes are also repressed by exogenous cytokinin [25]. A global analysis of gene expression events in rice showed that the expression changes caused by Pi starvation were reversed by exogenous cytokinin treatment. A large increase in intracellular phosphate levels may reduce the phosphate starvation signaling triggered by exogenous cytokinin [26]. CRE1, the receptor for cytokinin, has been found to participate in suppressing the regulation of many genes following Pi deficiency [22], [27]. This implies that a close crosstalk exists between Pi and the cytokinin signaling transduction pathway [23].

Recently, it has been reported that OsARF12 was involved in Pi homeostasis and that OsARF16 facilitated the efficient utilization of Pi in rice [28], [29]. However, the underlying mechanism linking auxin/cytokinin and Pi signaling is largely unknown. In this study, we analyzed the differences in Pi transport and Pi signaling between the *osarf16* mutant and NIP when subjected to cytokinin treatment. We found that OsARF16 was associated with cytokinin regulation of phosphate uptake and phosphate signaling in rice.

## Materials and Methods

### Plant materials and growth conditions

Rice plants (*Oryza sativa L.*) wild-type (WT) NIP and *osarf16* mutant (a knock-out mutant) [29] were grown in culture solution combined with phytohormone treatments in a glasshouse with a light∶ dark cycle of 12∶ 12 h at 30∶ 24°C (day∶ night) and 60–70% humidity, and the pH was adjusted to 5.8 with HCl. For hydroponics, seedlings were transferred to a plastic net floating on the nutrient solution containing 0.323 mM NaH_2_PO_4_ or without NaH_2_PO_4_ for Pi deficiency treatment (−Pi or +Pi). Phytohormone treatments were performed with 0.01 µM to 1 µM of 6-BA for 7days, respectively. The hydroponic experiments were performed in a standard rice culture solution containing 1.425 mM NH_4_NO_3_, 0.323 mM NaH_2_PO_4_, 0.513 mM K_2_SO_4_, 0.998 mM CaCl_2_, 1.643 mM MgSO_4_, 0.009 mM MnCl_2_, 0.075 mM (NH_4_)_6_Mo_7_O_24_, 0.019 mM H_3_BO_3_, 0.155 mM CuSO_4_, 0.036 mM FeCl_3_, 0.070 mM citric acid and 0.152 mM ZnSO_4_.

### β-glucuronidase (GUS) staining and analysis of GUS activity

The construction of *OsARF16* promoter–GUS was performed according to a published method [29]. Exactly 2641 bp of the OsARF16 promoter sequence upstream of its ATG were cloned in front of the GUS gene and was introduced into the *Agrobacterium tumefaciens* strain EHA105. To examine the progression of cell division, the transgenic plant carrying *pCYCB1;1:Dbox-GUS* was crossed with *osarf16* mutant and homozygous plants in the *osarf16* background were assayed for GUS staining [30]. GUS staining of seedlings was performed using 100 mM sodium phosphate buffer (pH 7.0) containing 0.1% v/vTriton X-100 and 2 mM X-Gluc (Sangon, Shanghai, China), and samples were incubated at 37°C overnight. Stained tissues were observed using a Carl Zeiss laser scanning system LSM510 (http://www.zeiss.com/) and a Leica MZ95 stereomicroscope (Leica Instrument, Nusslosh, Germany). The measurement of GUS activity was performed as described by Jefferson [31].

### QRT-PCR analysis

Total RNA was isolated from leaves or roots of 7-day-old seedlings. The methods for RNA extraction, reverse transcription and qRT-PCR were conducted as described in a previous report [29]. The sequences of the corresponding primers for qRT-PCR are listed in Table S1, S2, S3 and S4. *OsACTIN* was used as an internal standard to calculate the relative fold differences based on the comparative *Ct* method. 2^−ΔΔ*Ct*^ refers to the fold difference in expression of cytokinin-related genes under cytokinin treatment (3 hr) compared with the untreated seedlings. Heat map representation was performed using the normalized *Ct* value with Treeview 1.6 to visualize the analysis data. The different colors correspond to the values of the gene change-fold ratio shown in the bar. The data were analyzed by three independent repeats.

### Measurement of Pi contents

NIP and *osarf16* mutants were analyzed to determine their total P contents. P measurements were performed using Flow Analyser SAN++ (Skalar Analytical B.V., Breda, Netherlands). For Pi transport experiment, the rice plants were grown for 7days under Pi deficiency solution, and then were transferred to Pi supply solution (1 mM Pi) with or without cytokinin (6-BA). Long time Pi starvation depressed total Pi level in rice seedlings and resulted in activity of Pi transport during the first 24 hour periods after Pi resupply. For each sample in the experiment, 0.1 g tissue was dried at 80°C for 48 hr and digested with HNO_3_/H_2_O_2_ at 110°C for 0.5 hr using a microware 3000 digestor (Anton Paar, Graz, Austria). Five biological replicates were performed for each sample in all experiments.

### Statistics of root system parameters

The root system of rice plant was put into a container filled with distilled water. In order to minimize the intercross among the roots during image scanning, the whole root system was carefully separated into 5 portions, and each portion was transferred to individual container for scanning. The number of lateral roots was obtained by scanning. The average lengths of root hairs (3 mm from the root tips) were measured by a LEICA MZ95 stereomicroscope with camera scale (Leica Instrument, Nusslosh, Germany).

### Qualitative analysis of root-associated APase activity

Root APase staining was performed according to Bozzo's report [32]. The roots were excised from 7 days Pi-deprived and Pi-supplied seedlings and incubated with a 5-bromo-4-chloro-3-indolyl-phosphate (BCIP) content agar solution containing 50 mM sodium acetate (pH 5.5) with 10 mM MgCl_2_, 0.6% agar and 0.08% BCIP at room temperature for 20 min. The blue color on the root surface, formed by hydrolysis of BCIP, was photographed using an EOS 40D camera (Canon Corporation, Tokyo, Japan). Protein (1 µg) was used for APase activity assay, and the protein was added to 620 µl of reaction buffer (50 mM NaAc pH 5.5 and 10 mM MgCl_2_), and 10 µl of p-nitrophenol phosphate (10 mg ml^−1^ pNPP; Sigma). After incubation at 37°C for 10 min, the reaction was stopped by 1.2 ml of 1M NaOH, and then absorbance was measured at 412 nm wavelength. Phosphatase activity was counted as ng of pNPP accumulated µg^−1^ soluble protein min^−1^. These experiments were repeated three times.

## Results

### Physiological and morphological evidence that OsARF16 is involved in cytokinin responses in rice seedlings

**Figure 1:**
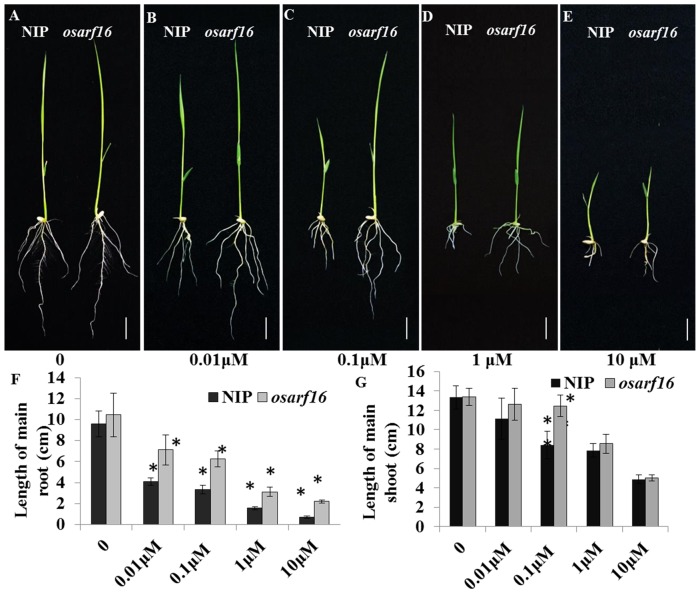
Physiological evidences for involvement of OsARF16 in cytokinin (6-BA) Responses. (A–E) Phenotype of NIP and *osarf16* mutant under different concentration of 6-BA treatments (0/0.01/0.1/1/10 µM) using 7-day-old seedlings (Bar represents 2 cm). The graphs represent statistics data of main root length (F) and main shoot length (G). Data are shown as the mean ± SD (n = 5). Significant (P<0.05) differences in length of main root and main shoot between *osarf16* and NIP are indicated by an asterisk.

In previous studies, we have reported that the OsARF16 is required for auxin signaling and the phosphate starvation response in rice. The phenotype of *osarf16* mutant under Pi deficiency condition was well studied in our last publication [29]. In the present study, the mutant *osarf16* was used to investigate how auxin response factor is involved in cytokinin signaling. To explore the possible effects of OsARF16 on morphological and physiological responses to cytokinin (6-BA) treatment, wild-type (NIP) and mutant *osarf16* seeds were grown in nutrient solutions containing different concentrations of 6-BA. The concentrations of 6-BA used in this experiment were 0, 0.01, 0.1, 1.0 and 10 µM. After 7 days of treatment, the root growth was considerably inhibited by 6-BA in the NIP seedlings (Fig. 1A–E). The root lengths of NIP declined significantly from 9.59 cm to 0.72 cm when the 6-BA concentration increased from 0 to 10 µM. Under the same conditions, the root growth inhibition was less significant in the *osarf16* mutant. As the 6-BA concentration increased, the *osarf16* mutant root lengths fell from 10.44 cm to 2.21 cm (Fig. 1F). In addition to root growth inhibition, shoot elongation was also affected by 6-BA application in NIP. The shoot lengths of NIP declined significantly from 13.32 cm to 4.87 cm when the 6-BA concentration increased from 0 to 10 µM. Under the same conditions, the root growth inhibition was less significant in the *osarf16* mutant. Specially, the shoot length in NIP was only 68% of that in the *osarf16* mutant under the 0.1 µM 6-BA treatment (Fig. 1C, G).

**Figure 2:**
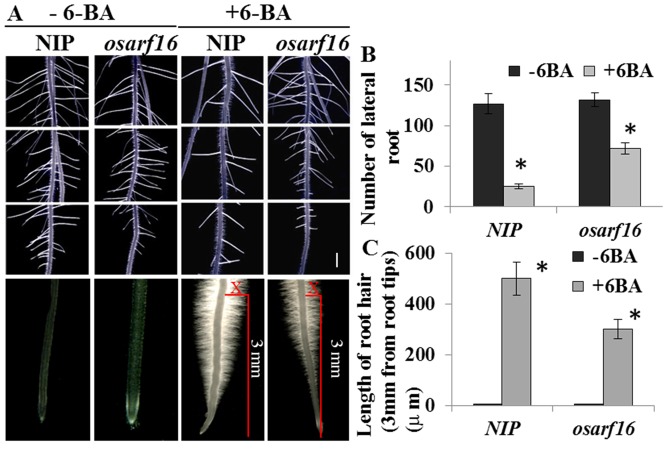
Effects of cytokinin on regulation of root system architecture (RSA). (A) 0.1 µM 6-BA treatment reduced the number of lateral root in primary root and induced root hair of NIP and *osarf16* mutant in primary root tips (The bar represents 5 mm). The graphs represent statistics data of lateral root number (B) and average root hairs length (C). Data are shown as the mean ± SD (n = 5). Significant (P<0.05) differences in number of lateral root in primary root and average length of root hairs in primary root tips between *osarf16* and NIP are indicated by an asterisk.

Cytokinin regulates root system architecture (RSA) by disturbing cell division activity and increasing the length of the root hairs at the root tips [33], [34]. In the present study, we measured the number of lateral roots on the main roots and the average lengths of the root hairs on the root tips of both NIP and the *osarf16* mutant under the 0.1 µM 6-BA treatment. The results showed that the number of lateral roots on *osarf16* was much greater than on NIP under 0.1 µM 6-BA treatment (Fig. 2A). Without 6-BA application, the number of lateral roots on NIP was similar to the *osarf16* mutant. The 0.1 µM 6-BA treatment reduced the number of lateral roots by 80% in the NIP seedlings. However, the reduction was only 45% in the *osarf16* mutant seedlings (Fig. 2B). The number of lateral roots in *osarf16* primary root was almost twice as much as NIP under 0.1 µM 6-BA treatment. When 6-BA was not applied, there were no root hairs on the root tips of both NIP and the *osarf16* mutant. On average, the lengths of the root hairs (3 mm from the root tips) could be increased to almost 500 µm on the tips of NIP, but only to 300 µm on the tips of the *osarf16* mutant under the 0.1 µM 6-BA treatment (Fig. 2C). The results revealed that the *osarf16* mutant was more insensitive to cytokinin treatment than NIP.

To confirm that the phenotypes described above were caused by the loss-of-function of *OsARF16*, we transformed the *OsARF16* gene back into the *osarf16* mutant and three independent complementation lines (*osarf16/C*) were chosen for analysis. The growth parameters for NIP and *osarf16/C* were approximately the same under the different 6-BA treatments (0, 0.01, 0.1, 1.0 and 10 µm) (Figure S1). Again, we used various cytokinins (kinetin, zeatin) to confirm the involvement of OsARF16 in the cytokinin responses. Kinetin and zeatin inhibited root system elongation and shoot growth and *osarf16* was, again, less sensitive to both cytokinins than *NIP* (Figure S2). The results confirmed that OsARF16 was involved in plant responses to cytokinin.

### OsARF16 affects cytokinin signaling

**Figure 3:**
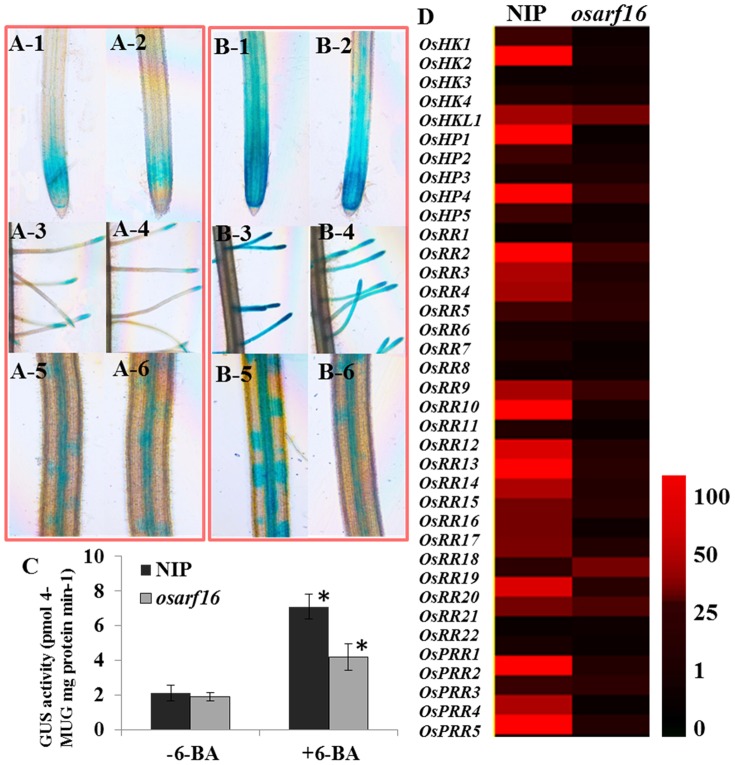
OsARF16 affects cytokinin signaling. (A, B) *pCYCB1;1:Dbox-GUS* staining was performed in primary root tips, lateral roots and lateral root primordia of NIP and *osarf16* mutant under −6-BA/+6-BA treatment. Concentration of 6-BA in the treatments was 0.1 µM. *pCYCB1;1:Dbox-GUS* staining in root tips of NIP (A-1) and *osarf16* mutant (A-2) under −6-BA treatment; *pCYCB1;1:Dbox-GUS* staining in lateral roots of NIP (A-3) and *osarf16* mutant (A-4) under −6-BA treatment; *pCYCB1;1:Dbox-GUS* staining in lateral root primordia of NIP (A-5) and *osarf16* mutant (A-6) under −6-BA treatment; *pCYCB1;1:Dbox-GUS* staining in primary root tips of NIP (B-1) and *osarf16* mutant (B-2) under +6-BA treatment; *pCYCB1;1:Dbox-GUS* staining in lateral roots of NIP (B-3) and *osarf16* mutant(B-4) under +6-BA treatment; *pCYCB1;1:Dbox-GUS* staining in lateral root primordia of NIP (B-5) and *osarf16* mutant (B-6) under +6-BA treatment. (C) Analysis of GUS activity. The entire root of the seedling was used for analysis. The graph represents statistics data of GUS activity in NIP and *osarf16* mutant roots. Data are shown as the mean ± SD (n = 5). Significant (P<0.05) differences in GUS activity between *osarf16* and NIP are indicated by an asterisk. (D) Analysis of the expression levels of cytokinin signaling related genes in *osarf16* mutant and NIP. The data of 2^−ΔΔ*Ct*^ (qRT data) refers to the fold difference in expression of cytokinin-related genes under cytokinin treatment (3 hr) compared with the untreated seedlings. Heat map representation was performed using the normalized 2^−ΔΔ*Ct*^ values with Treeview 1.6 to visualize the analysis data. The different colors correspond to the values of the gene change-fold ratio shown in the bar. The data were analyzed by three independent repeats.

To further determine the cytokinin signaling changes in the *osarf16* mutant, we analyzed cell division activity in the primary root tips, the lateral roots in primary root and the lateral root primordia under 6-BA treatment using the *pCYCB1;1:Dbox-GUS* reporter [30]. Five positive transgenic lines were used for this analysis. Under the control treatment, the cell division activities, as indicated by the expressions of the *pCYCB1;1:Dbox-GUS* reporter in NIP and the *osarf16* mutant, were almost the same. After 6-BA treatment (3 hr), the cell division activities in the primary root tips, the lateral roots in primary root and the lateral root primordia considerably increased in NIP, whereas the induction of cell division activity in *osarf16* was much lower than in NIP (Fig. 3A, B). The GUS activity data is shown in Fig. 3C. The GUS activity under −6-BA treatment in NIP was almost the same as that in *osarf16* mutant. However, the GUS activity in NIP was 69.0% higher than that in *osarf16* mutant. The data was essentially in agreement with the GUS staining results showed in Fig. 3 A and B.

Many studies have investigated cytokinin signaling in rice [35], [36]. We analyzed the expression of 37 cytokinin response genes in both NIP and the *osarf16* mutant in order to improve our understanding of the mechanism behind OsARF16 involvement in the cytokinin signaling system. These genes are all part of two-component regulatory systems. Five belong to HKs (cytokinin-response histidine protein kinase) (OsHK1–4, OsHKL1), five to HPs (histidine phosphotransfer proteins) (OsHP1–5), fifteen to type-A RRs (response regulators) (OsRR1–15), seven to type-B RR genes (OsRR16–22), and five are predicted pseudo-response regulators (OsPRR1–5) [37]. A qRT-PCR analysis was performed on the roots of 7-day-old NIP and *osarf16* seedlings grown in culture solutions with or without 6-BA (the primer sequences information is provided in Table S1). The results showed that all 37 cytokinin responsive genes were sharply induced by 6-BA treatment in the roots of the NIP seedlings. However, in the *osarf16* mutant seedlings, the induction of these cytokinin responsive genes was reduced under the 6-BA treatment compared to the NIP seedlings (Fig. 3D). This suggested that cytokinin signaling in *osarf16* was compromised compared to NIP.

### The expression pattern of *OsARF16* was dramatically changed by cytokinin treatment

**Figure 4:**
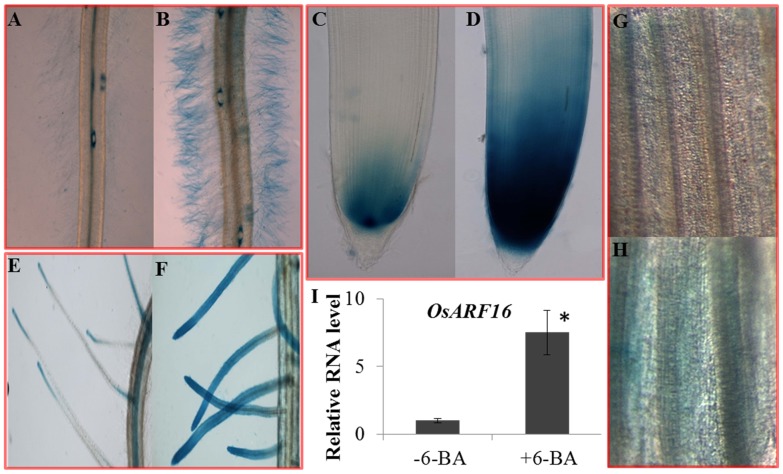
Analysis of *OsARF16* expression pattern under cytokinin treatment using *OsARF16*-Promoter: GUS lines. The expression pattern of *OsARF16* in primary root tips under (A) control and (B) 0.1 µM 6-BA treatment for 3 hr; The expression pattern of *OsARF16* in root hairs of primary root under (C) control and (D) 0.1 µM 6-BA treatment for 3 hr; The expression pattern of *OsARF16* in lateral roots of primary root under (E) control and (F) 0.1 µM 6-BA treatment for 3 hr. The expression pattern of *OsARF16* in leaves under (G) control and (H) 0.1 µM 6-BA treatment for 3 hr. (I) qRT-PCR method was used to confirm the *OsARF16* expression pattern changes in roots under 0.1 µM 6-BA treatment for 3 hr. Significant (P<0.05) differences in the expression level of *OsARF16* between 6-BA treatment and mock treatment is indicated by an asterisk.

We used the GUS reporter gene to evaluate the *OsARF16* expression patterns in the roots and shoots under −6-BA treatment and +6-BA treatment conditions. Five positive transgenic lines were used for this analysis. Under the control treatment (−6-BA treatment), *OsARF16* was expressed in root tips and stele of both primary roots (Fig. 4 A, C) and lateral roots in primary root (Fig. 4 E). Under +6-BA treatment (3 hr), the *OsARF16* expression level increased several times compared to the control treatment, especially in the root hairs (Fig. 4A–F). The *OsARF16* expression level was almost undetectable in root hairs under the control treatment, but was strongly induced by the +6-BA treatment. Meanwhile, the expression level of *OsARF16* was also induced by 6-BA treatment in shoots (Fig. 4G, H). We used qRT-PCR to confirm the *OsARF16* expression pattern changes in roots under the control and +6-BA treatment conditions and the results were consistent with the GUS staining analysis results (Fig. 4I). The expression level was 7.5 folds induced by 6-BA treatment in the whole root organ.

### Analysis of total phosphorus (P) content over a time-course in NIP and the *osarf16* mutant

**Figure 5:**
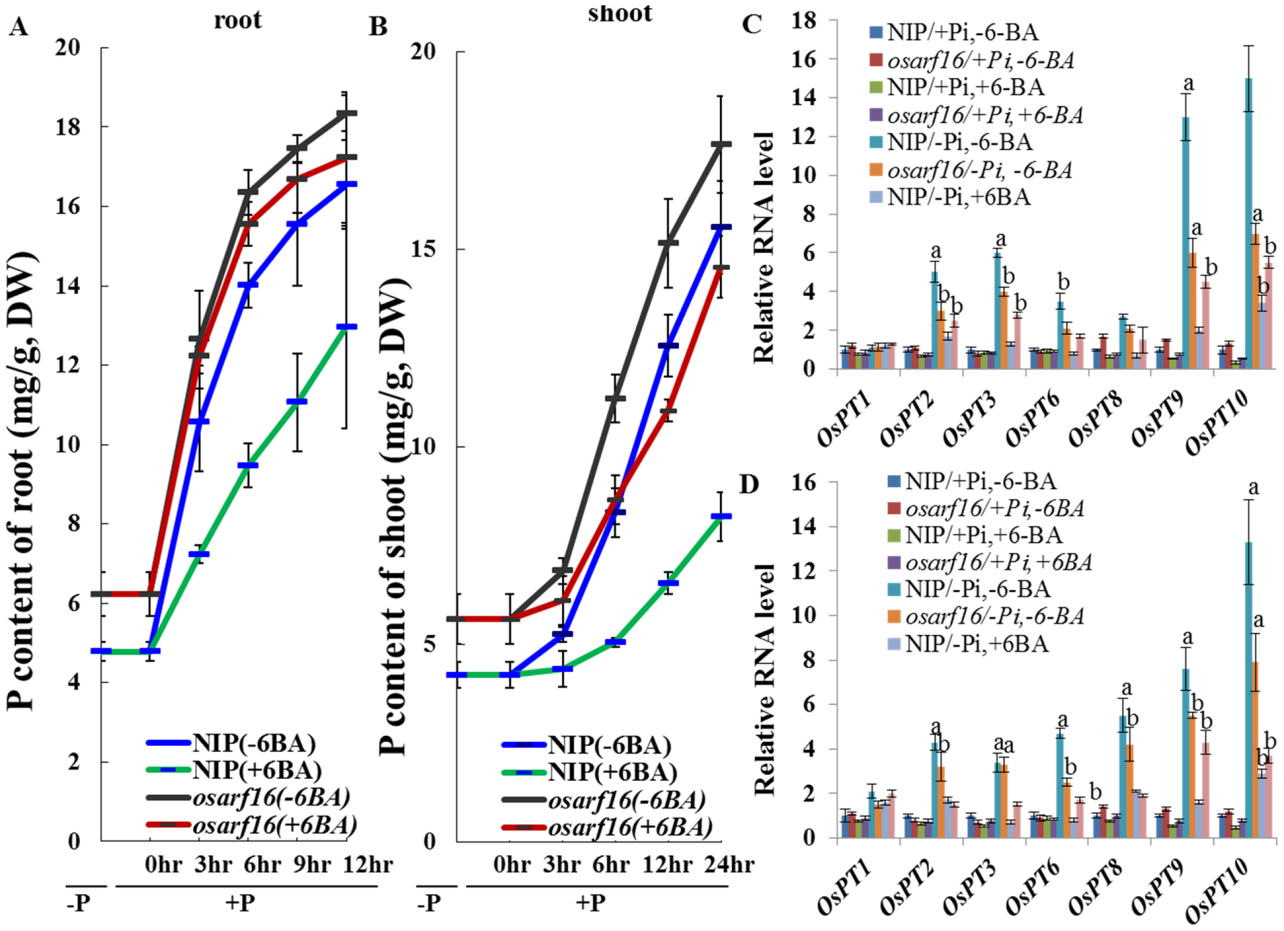
Analysis of total phosphorus (P) contents over a time-course in both NIP and *osarf16* mutant. (A,B) Total P contents in NIP and *osarf16* mutant after Pi resupply using 7-day-old Pi-deprived seedlings: (A) in roots and (B) in shoots. The statistics of P contents were calculated for five independent biological replications. (C, D) Expression of Pi transporter coding genes in Pi-sufficient (+Pi) and Pi-deficient (−Pi) treatments with or without 6-BA (C) in roots and (D) in shoots. Concentration of 6-BA in the treatments was 0.1 µM. The *ACTIN* gene of rice was used as the reference gene for qRT-PCR. Value ± SD of five independent replicates. “a” indicated significant difference in expression levels of *OsARF16* from treatments to mock at 1% by student's *t* test. “b” indicated significant difference in expression levels of *OsARF16* from treatments to mock at 5% by student's *t* test.

The total P contents over a time-course were measured in NIP and the *osarf16* mutant seedlings in order to clarify the function of OsARF16 in the cytokinin-mediated inhibition of phosphate transport between roots and shoots. The Pi-deprived rice seedlings, which were grown in a Pi free nutrient solution for 7 days, were transferred to a nutrient solution containing 1 mM Pi in order to monitor P contents in the shoots over a 24 h period and in roots over a 12 h period after Pi application. After 7 days of Pi starvation, the total P contents were only 4.7 mg g^−1^ in the NIP roots and 6.2 mg g^−1^ in the *osarf16* mutant roots. When resupplied with 1 mM Pi, the P contents in the roots rapidly increased in both NIP (16.55 mg g^−1^) and *osarf16* (18.35 mg g^−1^) after 12 h of Pi resupply. Under the same conditions, the P contents in the shoots were 4.2 mg g^−1^ in NIP and 5.6 mg g^−1^ in the *osarf16* mutant. When resupplied with 1 mM Pi, the P content only increased to 12.96 mg g^−1^ in NIP roots with 6-BA application (78.3% of the 6-BA free treatment) after 24 h of Pi resupply and increased to 17.23 mg g^−1^ in the *osarf16* shoots (93.4% of the 6-BA free treatment). On the other hand, the P contents in the shoots also rose in both NIP (15.56 mg g^−1^) and *osarf16* (17.65 mg g^−1^) after 24 h of Pi resupply. With 6-BA application, the P content only increased to 8.2 mg g^−1^ in NIP shoots (52.9% of the 6-BA free treatment) after 24 h of Pi resupply and increased to 14.5 mg g^−1^ in the *osarf16* shoots (82.9% of the 6-BA free treatment). Cytokinin (6-BA) clearly inhibited Pi transport in NIP, but the inhibition of Pi transport by cytokinin was less obvious in the *osarf16* mutant seedlings compared to NIP (Fig. 5A, B).

Furthermore, we analyze shoot/root ratios of P contents in both NIP and *osarf16* mutant under different conditions. After 7 days Pi starvation, the shoot/root ratios of P contents in both NIP and *osarf16* mutant were nearly 50%. When resupplied with 1 mM Pi, the shoot/root ratios of P contents were increased quickly after 3 hours Pi resupply and decreased to 50% gradually thereafter in both NIP and *osarf16* mutant. With 6-BA application, the shoot/root ratios of P contents increased to 62% after 3 hours Pi resupply and did not decreased obviously during 12 hour Pi resupply period in NIP. With 6-BA application, the shoot/root ratios of P contents increased to 65% after 3 hours Pi resupply and decreased obviously during 12 hour Pi resupply period in *osarf16* mutant (Figure S3). The 6-BA treatment affects the shoot/root ratios of P contents in NIP seedlings, but it was less obvious in the *osarf16* mutant.

### Involvement of OsARF16 in cytokinin-mediated inhibition of Pi transport

The *PHOSPHATE TRANSPORTER1* (*PHT1*) gene family plays a number of critical roles in Pi uptake, translocation and homeostasis in *Arabidopsis*. Some of these genes have also been identified in rice [38], [39]. In this study, we used qRT-PCR to analyze the expression levels of several members of the rice *PHT1* gene family (including *OsPT1/2/3/6/8/9* and *10*). The primer sequences information is provided in Table S2. Most of these phosphate transporter coding genes were up-regulated by Pi deficiency and their expression levels were inhibited in NIP roots and shoots by applications of 0.1 µM 6-BA (Fig. 5C, D). The expression levels of these *OsPTs* were higher in the *osarf16* mutant than in NIP under −Pi/+6-BA treatments.

### Expression analysis of *phosphate (Pi) starvation-induced* (*PSI*) genes in NIP and the *osarf16* mutant

**Figure 6:**
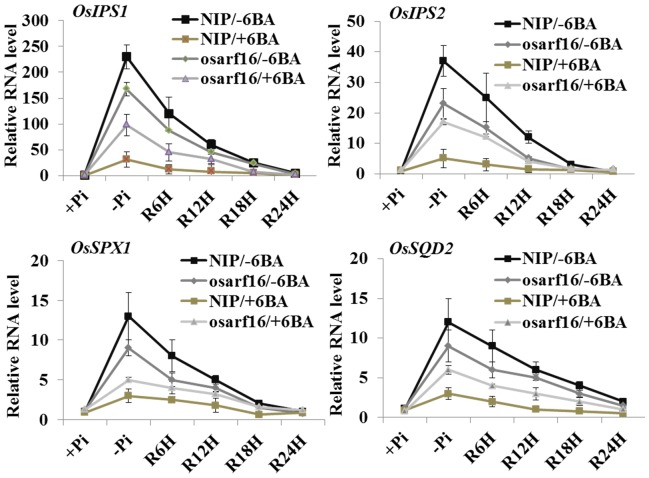
Expression analysis of *phosphate (Pi) starvation-induced* (*PSI*) genes, *OsIPS1*, *OsIPS2*, *OsSPX1*, *OsSQD2* in NIP and *osarf16* mutant. Analysis of expressions were performed under phosphate-sufficient (+Pi), phosphate-deficient (−Pi) and Pi resupply conditions (R6h, R12h, R18h and R24h refer to resupply times of 6, 12, 18 and 24 h, respectively) with or without 6-BA treatments. Concentration of 6-BA in the treatments was 0.1 µM. 7-day-old seedlings of NIP and *osarf16* mutant were used for quantitative reverse transcription polymerase chain reaction (qRT-PCR) analysis. Value ± SD of five independent replicates.

To test whether the *phosphate (Pi) starvation-induced* (*PSI*) genes were also affected by cytokinin treatment, we chose four genes: *OsIPS1*/*OsIPS2*, *OsSPX1* [40] and *OsSQD2* [41]. Then we analyzed the transcript abundance in NIP and the *osarf16* mutant and under +Pi/−6-BA, −Pi/−6-BA, +Pi (resupply)/−6-BA, +Pi/+6-BA, −Pi/+6-BA and +Pi (resupply)/+6-BA treatments (The primer sequences information are provided in Table S3). The expression level analysis revealed that these four genes were sharply up-regulated under the −Pi/−6-BA treatment and quickly fell down to background levels during the 24 h period after Pi resupply (Fig. 6). All four *PSI* genes were repressed by 6-BA application under the −Pi treatment in NIP. For example, *OsIPS1* was 230 times higher under the −Pi/−6-BA treatment compared to the +Pi/−6-BA treatment, but was only 25 times higher under −Pi/+6-BA treatment. In the *osarf16* mutant, the expression levels of these *PSI* genes were much higher than that in NIP under the −Pi/+6-BA treatment.

### Analysis of acid phosphatase (APase) activity and expression of six *PAPase* genes

**Figure 7:**
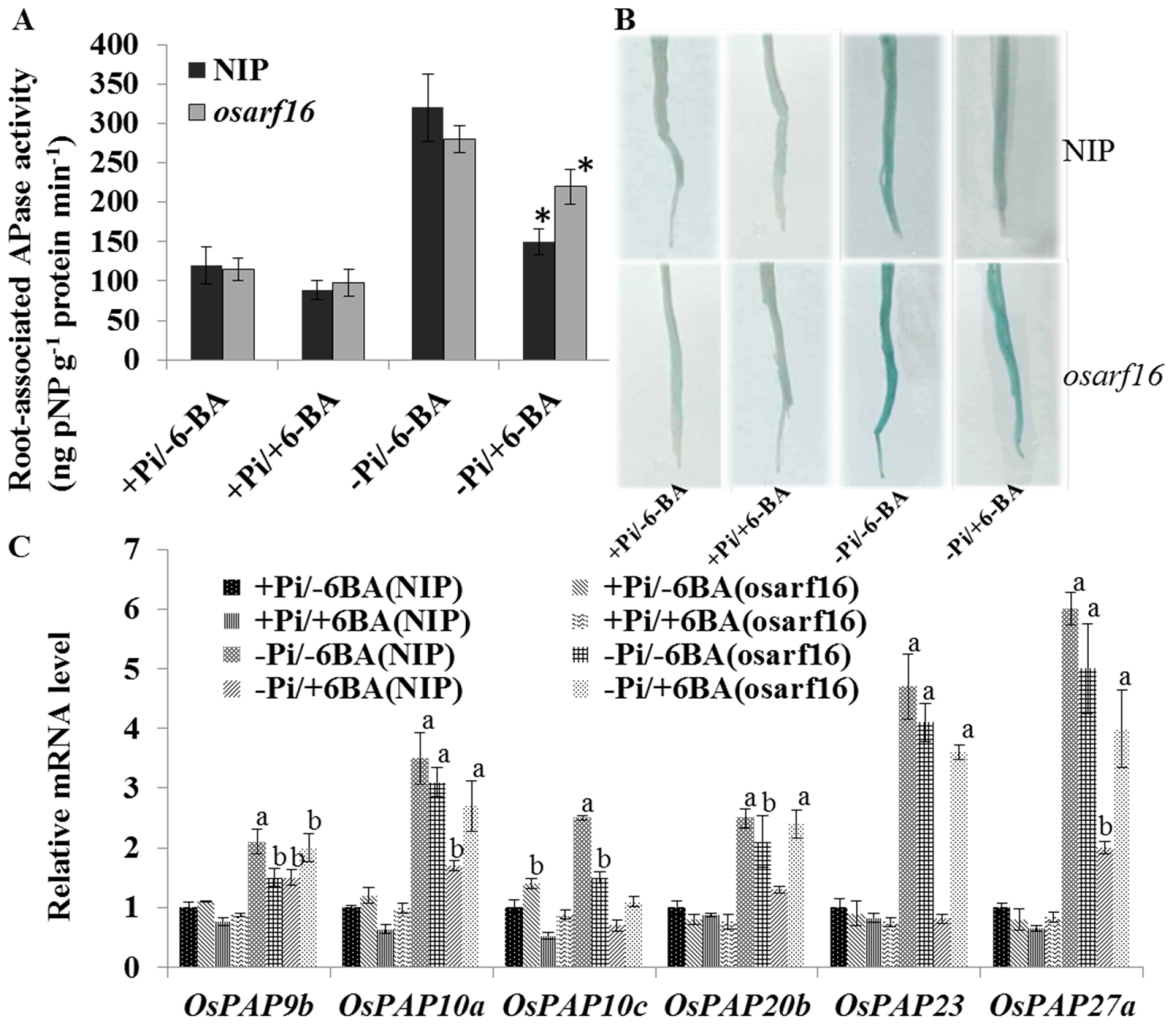
Analysis of acid phosphatase (APase) activity and expression of six purple APase coding genes. (A) Acid phosphatase (APase) activity in NIP and *osarf16* mutant under +Pi/−6BA, +Pi/+6BA, −Pi/−6BA and −Pi/+6BA conditions respectively. Concentration of 6-BA in the treatments was 0.1 µM. The graph represents statistics data of root-associated APase activity. (B) Acid phosphatase (APase) activity in the root surface of NIP and *osarf16* mutant under +Pi/−6BA, +Pi/+6BA, −Pi/−6BA and −Pi/+6BA conditions respectively. (C) Expression levels of six purple APase coding genes under +Pi/−6BA, +Pi/+6BA, −Pi/−6BA and −Pi/+6BA conditions respectively. Data are shown as the mean ± SD (n = 5). Significant (P<0.05) differences in APase activity between *osarf16* mutant and NIP are indicated by an asterisk. “a” indicated significant difference in expression levels of *OsPAPs* from treatments to mock at 1% by student's *t* test. “b” indicated significant difference in expression levels of *OsPAPs* from treatments to mock at 5% by student's *t* test.

The induction of APase activity under Pi deficiency conditions helps to catalyze inorganic phosphate hydrolysis from organophosphates and is an important strategy that enables plants to deal with Pi deficiency [42]. The expressions of *PAPase* genes and APase activity were measured in NIP and the *osarf16* mutant in order to understand how OsARF16 is involved in Pi homeostasis through its effects on APase activity. Rice seedlings were grown under +Pi/−6BA, +Pi/+6BA, −Pi/−6BA and −Pi/+6BA conditions. In contrast, 6-BA application significantly suppressed the APase activity in NIP under Pi deficiency conditions (−Pi/+6-BA treatment). However, the APase activity was 50% higher in the *osarf16* mutant than in NIP the under −Pi/+6-BA treatment (Fig. 7A). The data were gained from the analysis of root intracellular APase activity on substrate pNPP (Fig. 7B). Many purple APase (PAPase) genes, which are involved in Pi uptake and Pi starvation responses, have been reported by Wang *et al.* (2009). We chose six of the PAPase genes for this experiment (*OsPAP9b*, *OsPAP10a*, *OsPAP10c*, *OsPAP20b*, *OsPAP23* and *OsPAP27a*) because they were induced by −Pi deficiency by more than two-fold (The primer sequences information is provided in Table S4). Notably, all six genes were down-regulated by 6-BA treatment under the −Pi deficiency treatment in NIP. The expression levels of these genes were higher in the *osarf16* mutant than in NIP under the −Pi/+6-BA treatment (Fig. 7C). This suggested that cytokinin-mediated inhibition of *APase* expression is dependent on OsARF16.

## Discussion

### OsARF16 is a key regulator of the cytokinin response in rice

ARFs are involved in many important biological functions, including plant development and nutritional homeostasis [8], [43]. OsARF16 as a transcriptional activator that is highly homologous to AtARF7/19 in *Arabidopsis* and is implicated in Pi deficiency response related root system architecture changes [29], [44]. In *Arabidopsis* meristems, cytokinin controls the differentiation rate of transit-amplifying cells by antagonizing the plant hormone auxin, which is needed to sustain cell proliferation [15]. In our previous studies, we identified the *osarf16* mutant and characterized its biological effects on auxin signaling and auxin distribution [29]. However, how ARFs interact with cytokinin signaling in rice is largely unknown.

Cytokinin suppresses the growth of rice seedlings and *OsARF16* loss-of-function attenuates sensitivity to exogenous cytokinin treatment (Fig. 1). High concentration 6-BA treatments cause severe growth defects in wild-type seedlings, including root system development and shoot elongation. In particular, there was an induction in lateral root numbers and main root growth in the *osarf16* mutant compared to NIP under 6-BA treatment. This suggested that OsARF16 was required for cytokinin responses in rice.

In rice plants, *PINs*, and *LAXs* are the two major gene families responsible for auxin transport *in vivo* [29]. Exogenous cytokinin treatment also inhibited the expression of auxin transport genes and disrupted auxin distribution, which is required for many of the physiological responses to cytokinin [45]. Our data showed that 6-BA treatment clearly down-regulated the expression levels of *OsPINs* and *OsLAXs* in NIP. However, the expression levels of the *OsPIN* and *OsLAX* family genes were much higher in the *osarf16* mutant than in NIP under the 6-BA treatment (Figure S4). OsARF16 may be involved in cytokinin responses by regulating the expression of auxin transporter coding genes.

### OsARF16 is involved in cytokinin-mediated negative regulation of phosphate uptake and phosphate signaling in rice (*Oryza sativa L.*)

Cytokinin inhibits phosphate transport by regulating the expression of the phosphate transporter coding genes [22], [23]. In this study we used 7-day-old Pi-deprived seedlings to investigate how OsARF16 is involved in the negative regulation of Pi uptake and transport by cytokinin. When transferred to the Pi supply nutrient solution, the phosphate contents in NIP seedlings rapidly rose (Fig. 5A) and cytokinin application could significantly block this process. However, in the *osarf16* mutant, the effect of cytokinin on preventing Pi transport from the roots to the shoots was weaker than in NIP (Fig. 5B). The expression levels of *OsPTs* were much higher in the *osarf16* mutant than that in NIP under the 6-BA treatment, which was consistent with the results of the Pi content measurements. These results indicated that OsARF16 was involved in cytokinin-mediated inhibition of Pi transport from the roots to the shoots by regulating *PT* expressions in rice.

### OsARF16 is an important transcription factor involved in the crosstalk between cytokinin and Pi starvation signaling

It has been already reported that Pi deficiency signaling could be inhibited by cytokinin and that there was a crosstalk between cytokinin and Pi deficiency signaling [25]. Cytokinin may repress the Pi starvation response by increasing intracellular phosphate content. Under Pi deficiency condition, more organic Pi is released into cellular Pi pool (inorganic Pi) driven by exogenous 6-BA [26]. In our experiment, total Pi (organic Pi and inorganic Pi) have been measured in NIP and *osarf16* mutant under different conditions. After 7 days Pi starvation, the Pi level in rice seedlings was greatly depressed and the increased of total Pi content after Pi resupply was inhibited by 6-BA treatment (Fig. 5). 6-BA treatment elevates inorganic Pi content by reducing organic Pi concentration (major component of total Pi). However, the full physiological and molecular relevance of how cytokinin is involved in Pi signaling remains unclear. In this study, we discovered that OsARF16, an auxin response factor, was an important regulator involved in the crosstalk between cytokinin and Pi deficiency signaling. Pi signaling was repressed by cytokinin treatment and it depended on a functioning OsARF16 gene. Under Pi deficiency conditions, the expressions of *OsIPS1/2*, *OsSPX1* and *OsSQD2* were inhibited by 6-BA treatment in NIP. However, inhibition of *PSI* gene expressions by 6-BA treatment in the *osarf16* mutant was compromised compared to the NIP seedlings.

Acid phosphatase activity was also reduced by cytokinin treatment. An increase in APase activity in the roots is a typical Pi starvation response when plants are subjected to Pi deficiency. Induction and secretion of acid phosphatases (APases) is a highly efficient mechanism that helps plants to survive and grow under Pi deficient conditions [28], [46], [47]. According to the results (Fig. 7A, B), the APase activity in NIP roots was reduced by 6-BA treatment under Pi deficiency conditions. The APase activity was much stronger in the *osarf16* mutant roots compared to the NIP roots under the same conditions. The expression level of some purple APase coding genes, such as *OsPAP20b*, *23* and *27a* in the *osarf16* mutant under the −Pi/+6-BA treatment, were almost twice than in NIP. These results strongly demonstrated that OsARF16 is involved in the crosstalk between cytokinin and Pi starvation signaling.

## Conclusion

In summary, we found that OsARF16 was involved in cytokinin-mediated inhibition of phosphate transport and phosphate deficiency signaling in rice. Our data show that OsARF16 plays a critical role in auxin and Pi starvation responses and is involved in the crosstalk between cytokinin and Pi deficiency signaling. Our next study will focus on the direct downstream targets of OsARF16 in order to reveal how cytokinin regulates Pi transport and Pi signaling in rice.

## Supporting Information

Figure S1 **Physiological results for** ***osarf16/C*** **under 6-BA treatment.**
(DOCX)

Figure S2 **Physiological evidence for OsARF16 was involved in various cytokinins (kinetin, zeatin) Responses.**
(DOCX)

Figure S3 **Analysis of shoot/root ratios of P contents in both NIP and** ***osarf16*** **mutant.**
(DOCX)

Figure S4 **The expression levels of** ***OsPIN*** **and** ***OsLAX*** **family genes.**
(DOCX)

Table S1 **The RT primer sequences 37 cytokinin response genes.**
(DOCX)

Table S2 **Primer sequences for phosphorus transporters.**
(DOCX)

Table S3 **Primer sequences for the Phosphate Starvation Induced genes (** ***PSIs*** **).**
(DOCX)

Table S4 **Primer sequences for purple acid phosphatase genes.**
(DOCX)
