# Supplementary material for: OsARF16 Is Involved in Cytokinin-Mediated Inhibition of Phosphate Transport and Phosphate Signaling in Rice (Oryza sativa L.)
Source: PLoS One. 2014 Nov 11;9(11):e112906. doi: 10.1371/journal.pone.0112906 (PMC4227850; doi:10.1371/journal.pone.0112906)
Supplement: Figure S1 — Physiological results for osarf16/C under 6-BA treatment. (DOCX) [file pone.0112906.s001.docx]

Figure S1


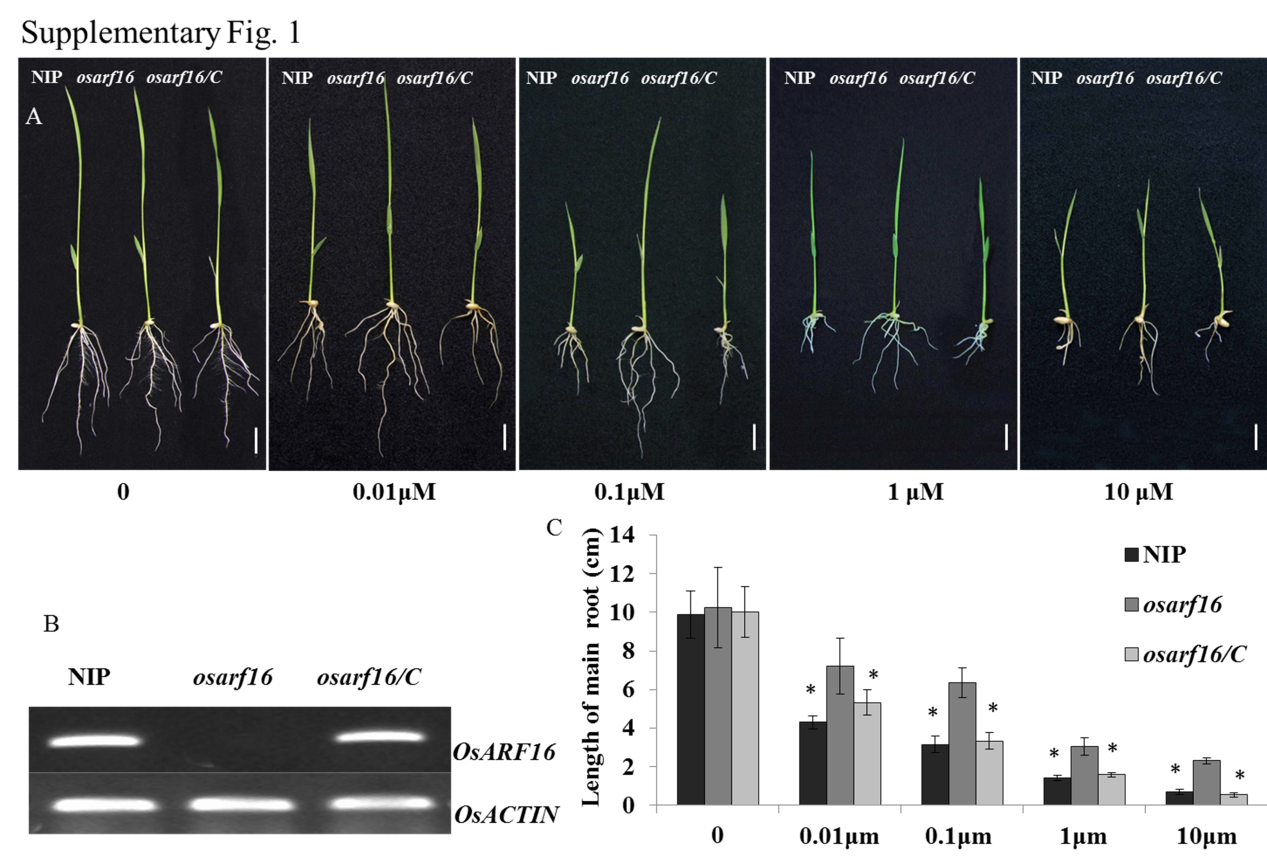


Figure S1 Morphological results for *osarf16/C* under 6-BA treatment. (A) Phenotype of NIP, *osarf16* and *osarf16/C* under different concentration 6-BA treatment (0/0.01/0.1/1/10μm). (Bar represents 2cm) (B) RT-PCR to confirm the mutant and mutant complementation line (*osarf16/C*). (C) The graph represents statistics data of main root length in NIP, *osarf16* and *osarf16/C* under different concentration 6-BA treatment. Data are shown as the mean ± SD (n = 5). Signiﬁcant (P < 0.05) differences in length of main roots between *osarf16* and NIP are indicated by an asterisk.
