# Supplementary material for: OsARF16 Is Involved in Cytokinin-Mediated Inhibition of Phosphate Transport and Phosphate Signaling in Rice (Oryza sativa L.)
Source: PLoS One. 2014 Nov 11;9(11):e112906. doi: 10.1371/journal.pone.0112906 (PMC4227850; doi:10.1371/journal.pone.0112906)
Supplement: Figure S2 — Physiological evidence for OsARF16 was involved in various cytokinins (kinetin, zeatin) Responses. (DOCX) [file pone.0112906.s002.docx]

Figure S2


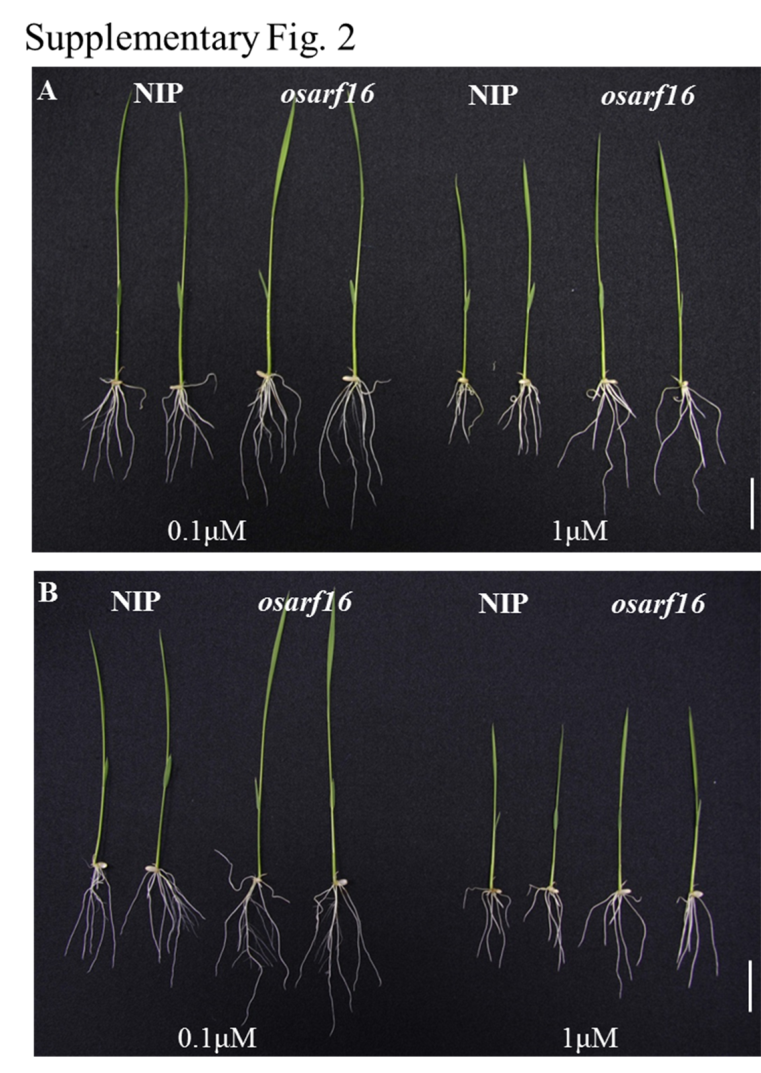


Figure S2 Physiological evidence for OsARF16 was involved in various cytokinins (kinetin, zeatin) Responses. (A) Phenotype of NIP and *osarf16* under kinetin treatments (from 0.1μM to 1μM) (B) Phenotype of NIP and *osarf16* under zeatin treatments (from 0.1μM to 1μM). (Bar represents 2cm).
