# Supplementary material for: OsARF16 Is Involved in Cytokinin-Mediated Inhibition of Phosphate Transport and Phosphate Signaling in Rice (Oryza sativa L.)
Source: PLoS One. 2014 Nov 11;9(11):e112906. doi: 10.1371/journal.pone.0112906 (PMC4227850; doi:10.1371/journal.pone.0112906)
Supplement: Figure S3 — Analysis of shoot/root ratios of P contents in both NIP and osarf16 mutant. (DOCX) [file pone.0112906.s003.docx]

Figure S3


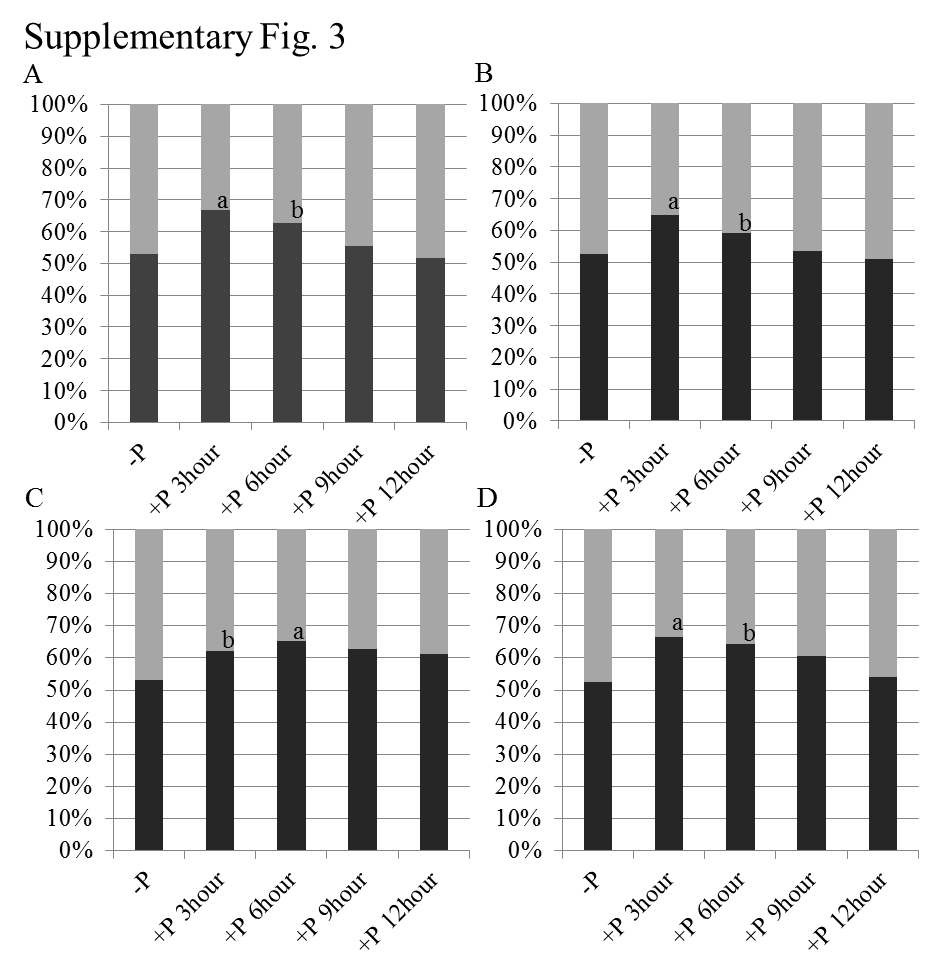


Figure S3 Analysis of shoot/root ratios of P contents in both NIP and *osarf16* mutant. (A) The shoot/root ratios of P contents in both NIP (A) and *osarf16* mutant (B) without 6-BA treatment. The shoot/root ratios of P contents in both NIP (C) and *osarf16* mutant (D) with 6-BA treatment. “a” indicated significant difference in expression levels of *OsPAPs* from treatments to mock at 1% by student’s *t* test. “b” indicated significant difference in expression levels of *OsPAPs* from treatments to mock at 5% by student’s *t* test.
