# Supplementary material for: OsARF16 Is Involved in Cytokinin-Mediated Inhibition of Phosphate Transport and Phosphate Signaling in Rice (Oryza sativa L.)
Source: PLoS One. 2014 Nov 11;9(11):e112906. doi: 10.1371/journal.pone.0112906 (PMC4227850; doi:10.1371/journal.pone.0112906)
Supplement: Figure S4 — The expression levels of OsPIN and OsLAX family genes. (DOCX) [file pone.0112906.s004.docx]

Figure S4


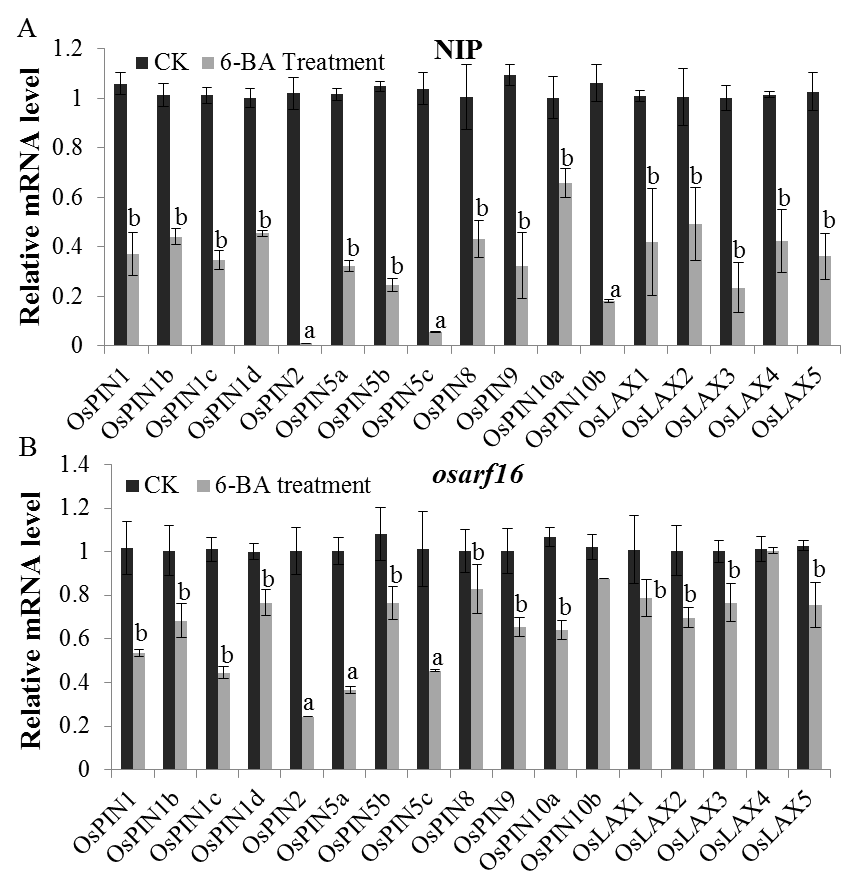


Figure S4 The expression levels of *OsPIN* and *OsLAX* family genes in NIP (A) and *osarf16* (B) mutant under different conditions. Data are shown as the mean ± SD (n = 5). “a” indicated significant difference in expression levels of *OsPAPs* from treatments to mock at 1% by student’s *t* test. “b” indicated significant difference in expression levels of *OsPAPs* from treatments to mock at 5% by student’s *t* test.
