# Supplementary material for: OsARF16 Is Involved in Cytokinin-Mediated Inhibition of Phosphate Transport and Phosphate Signaling in Rice (Oryza sativa L.)
Source: PLoS One. 2014 Nov 11;9(11):e112906. doi: 10.1371/journal.pone.0112906 (PMC4227850; doi:10.1371/journal.pone.0112906)
Supplement: Table S1 — The RT primer sequences 37 cytokinin response genes. (DOCX) [file pone.0112906.s005.docx]

| **Table S1** The RT primer sequences 37 cytokinin response genes | | |
| --- | --- | --- |
| OsHK1 | LOC_Os02g50480 RT U | TATGCTCAGACAGCCCAGAT |
| OsHK1 | LOC_Os02g50480 RT L | AGGCAGACTCGCACAAATAC |
| OsHK2 | LOC_Os10g21810 RT U | GATGATAATCCAGCCCACAG |
| OsHK2 | LOC_Os10g21810 RT L | CAACTATTCCAAGGGAAGCA |
| OsHK3 | LOC_Os01g69920 RT U | CCATGTCAGCGTGCTCAACT |
| OsHK3 | LOC_Os01g69920 RT L | TGCGTCGTGTCCAAATCAGT |
| OsHK4 | LOC_Os03g50860 RT U | CAACCCAGAGGGATTATGCA |
| OsHK4 | LOC_Os03g50860 RT L | TGATCGAGTTTCCCACCAAG |
| OsHKL1/OsCRL4 | LOC_Os12g26940 RT U | AGGGAGTTCAAGGCAGAGGT |
| OsHKL1/OsCRL4 | LOC_Os12g26940 RT L | TGGTGGAGGTAGAGCAAAGC |
| OsHP1 | LOC_Os09g39400 RT U | CCATGTTCTCCCAGGGTTTG |
| OsHP1 | LOC_Os09g39400 RT L | AATGCCATAAGGCACCCATC |
| OsHP2 | LOC_Os08g44350 RT U | AACGCCCTCGTCAACAAC |
| OsHP2 | LOC_Os08g44350 RT L | AAAGTCTTGAGGCACCCATC |
| OsHP3 | LOC_Os01g54050 RT U | ATTTGCGTCGCCAAGCTG |
| OsHP3 | LOC_Os01g54050 RT L | ATCCTAGAAGCGCCGATGC |
| OsHP4 | LOC_Os05g44570 RT U | CTTCGATCAGGGTTACTTGG |
| OsHP4 | LOC_Os05g44570 RT L | TGCTCCCTCTTCACTTTCTG |
| OsHP5 | LOC_Os05g09410 RT U | AGTTGCGGCTCTGTTCTTCA |
| OsHP5 | LOC_Os05g09410 RT L | ATGATTCCAGCTTCTGCTTCAG |
| OsRR1 | LOC_Os04g36070 RT U | GACGTACAGCGTCTCAGGAA |
| OsRR1 | LOC_Os04g36070 RT L | AAGCAGCCGTTTGACCAT |
| OsRR2 | LOC_Os02g35180 RT U | TGCTGGTGGTGGACGACTC |
| OsRR2 | LOC_Os02g35180 RT L | AACGCCTTGATGGCTTTGA |
| OsRR3 | LOC_Os02g58350 RT U | TGGAGGTTCTCAGCCTGGAT |
| OsRR3 | LOC_Os02g58350 RT L | CATTTCATGATGACGCGGTT |
| OsRR4 | LOC_Os01g72330 RT U | CACTGTGGATTCTGGGAGCA |
| OsRR4 | LOC_Os01g72330 RT L | TCAGCGAGCTTGACAGGTTT |
| OsRR5 | LOC_Os04g44280 RT U | TCATCTCCGGCATCCTCC |
| OsRR5 | LOC_Os04g44280 RT L | CTCCGCTCCTTCCTCCAA |
| OsRR6 | LOC_Os04g57720 RT U | AGGGTGACCACGGTGGAGT |
| OsRR6 | LOC_Os04g57720 RT L | GTACGGGCTTGAGCAGGAAG |
| OsRR7 | LOC_Os07g26720 RT U | AAGAGTGTCGTCGTCAAGGG |
| OsRR7 | LOC_Os07g26720 RT L | CTCCGGCATCCAGTAGTCC |
| OsRR8 | LOC_Os08g28950 RT U | TGATCGCCATGTTGTTTCC |
| OsRR8 | LOC_Os08g28950 RT L | CTCCTCCATCCAAGCACTTT |
| OsRR9 | LOC_Os11g04720 RT U | TGTCCTGGCTGTGGATGAT |
| OsRR9 | LOC_Os11g04720 RT L | CACAACTGGGATGTCCCTTA |
| OsRR10 | LOC_Os12g04500 RT U | TGAGGACAGCCCAATTTCTA |
| OsRR10 | LOC_Os12g04500 RT L | GGTGTTGTTCCTGCTGGTAG |
| OsRR11 | LOC_Os02g42060 RT U | GCTGTGGATAGCGGGAAGA |
| OsRR11 | LOC_Os02g42060 RT L | GTAGCACGCGGCTGAAGA |
| OsRR12 | LOC_Os08g26990 RT U | ATAACGTCCGAGTGACTGCA |
| OsRR12 | LOC_Os08g26990 RT L | GAATACGAGGCACATCAGCA |
| OsRR13 | LOC_Os04g13480 RT U | AACCGCATGATCCTGTCG |
| OsRR13 | LOC_Os04g13480 RT L | GCGCTGCTCTTGTTCTTCTT |
| OsRR14 | LOC_Os03g53100 RT U | AGGAGATCAATAGGGTCGTGG |
| OsRR14 | LOC_Os03g53100 RT L | CCTTGGACAGAGGCTTGGA |
| OsRR15 | LOC_Os08g28900 RT U | ATGTTGATCGCCATGTTGTT |
| OsRR15 | LOC_Os08g28900 RT L | TATAATCCTTTGCTCCTCCATC |
| OsRR16 | LOC_Os01g67770 RT U | CCTTGCCTGTGCCAGATAAA |
| OsRR16 | LOC_Os01g67770 RT L | ACTAGGGCCAACTGGGTGTT |
| OsRR17 | LOC_Os02g08500 RT U | AGAAGAAGCCAAGGGTCGTG |
| OsRR17 | LOC_Os02g08500 RT L | TGTTGTTTGCCTGGGTATGG |
| OsRR18 | LOC_Os02g55320 RT U | TGGGTGGATTTGAAGGACTA |
| OsRR18 | LOC_Os02g55320 RT L | CAGAAAGGATGGTGGACAGA |
| OsRR19 | LOC_Os03g12350 RT U | GTGGACGACGACCCTACATG |
| OsRR19 | LOC_Os03g12350 RT L | CCTCCATTCTGACGGGTTTA |
| OsRR20 | LOC_Os06g08440 RT U | GCCCAGAGTTGTATGGTCAG |
| OsRR20 | LOC_Os06g08440 RT L | AAGATGGAAGAGCAGCAGAA |
| OsRR21 | LOC_Os06g43910 RT U | CACAATGGCGGTTACAACG |
| OsRR21 | LOC_Os06g43910 RT L | ATGCCTGTTTGAGTGCTGATC |
| OsRR22/Ehd1 | LOC_Os10g32600 RT U | GCACATATTCCGAAAGCAAA |
| OsRR22/Ehd1 | LOC_Os10g32600 RT L | CTTCTCCGAGGTGGTTCACT |
| OsPRR1 | LOC_Os05g32890 RT U | TCGCCAGTAGCAACAAATCC |
| OsPRR1 | LOC_Os05g32890 RT L | AGGTTGAAGCCGTCTATCCC |
| OsPRR2 | LOC_Os04g28120 RT U | AGTGAAACGCCAGGACCTTC |
| OsPRR2 | LOC_Os04g28120 RT L | GACCCTTCAGCAGCAGCAGT |
| OsPRR3 | LOC_Os04g28160 RT U | AGCACTTGTGCCAATGAAAT |
| OsPRR3 | LOC_Os04g28160 RT L | GCAGGCTGATTAGTGTAGGG |
| OsPRR4 | LOC_Os04g28130 RT U | CCCTGCTGGTAGGCTTAGTG |
| OsPRR4 | LOC_Os04g28130 RT L | TCCAAGTTTGACGCACCTCT |
| OsPRR5 | LOC_Os05g32880 RT U | TCTGGACGGCCTATTCATTC |
| OsPRR5 | LOC_Os05g32880 RT L | ATTGCTCATCGCACTTCCTC |
