# Supplementary material for: OsARF16 Is Involved in Cytokinin-Mediated Inhibition of Phosphate Transport and Phosphate Signaling in Rice (Oryza sativa L.)
Source: PLoS One. 2014 Nov 11;9(11):e112906. doi: 10.1371/journal.pone.0112906 (PMC4227850; doi:10.1371/journal.pone.0112906)
Supplement: Table S2 — Primer sequences for phosphorus transporters. (DOCX) [file pone.0112906.s006.docx]

| **Table S2** Primer sequences for phosphorus transporters | |
| --- | --- |
| OsPT1 RT U: | CGCTTCCGTACGAGTGGTAGT |
| OsPT1 RT L: | GGTTCTTTCAAATCCAGGGAAA |
| OsPT2 RT U: | CACAAACTTCCTCGGTATGCT |
| OsPT2 RT L: | GAAACCCCACAAATCCACAAC |
| OsPT3 RT U: | AGACGGTGGTTCAACAGAG |
| OsPT3 RT L: | GCAAACCAAACTAACAAAATACC |
| OsPT6 RT U: | GCCCCTGCAAACTGTACTG |
| OsPT6 RT L: | AGCCAGGCCAGTTATATATCAAC |
| OsPT8 RT U: | CCTACTTGTGTTTGTCTATGTG |
| OsPT8 RT L: | GTGCCAAATTGCTGGTCTG |
| OsPT9 RT U: | CATAGGCTTGTCATCCTTTGG |
| OsPT9 RT L: | CACTGTAAATAAATCCGCGTTTC |
| OsPT10 RT U: | GAGCTCGCACCTCAGCAT |
| OsPT10 RT L: | GAGTTCACTCACACGGAGACC |
| OsACTIN RT U: | GGAACTGGTATGGTCAAGGC |
| OsACTIN RT U: | AGTCTCATGGATACCCGCAG |
