# Supplementary material for: OsARF16 Is Involved in Cytokinin-Mediated Inhibition of Phosphate Transport and Phosphate Signaling in Rice (Oryza sativa L.)
Source: PLoS One. 2014 Nov 11;9(11):e112906. doi: 10.1371/journal.pone.0112906 (PMC4227850; doi:10.1371/journal.pone.0112906)
Supplement: Table S3 — Primer sequences for the Phosphate Starvation Induced genes ( PSIs ). (DOCX) [file pone.0112906.s007.docx]

| **Table S3** Primer sequences for the Phosphate Starvation Induced genes (PSIs) | |
| --- | --- |
| OsIPS1 RT U: | AAGGGCAGGGCACACTCCACATTA |
| OsIPS1 RT L: | ATTAGAGCAAGGACCGAAACACA |
| OsIPS2 RT U: | CCTTCTTCTGGATTCCTCTC |
| OsIPS2 RT L: | AGTTCACCACAAAAGATACAGTAG |
| OsSPX1 RT U: | GACCAGCTTCTACCATCAAACG |
| OsSPX1 RT L: | AGTCTCAGGATACCCGCAG |
| OsSPX1 RT U: | CTGAAAACGGTAATGGATAGG |
| OsSPX1 RT L: | AACAACAACAGCACGAGC |
