# Supplementary material for: OsARF16 Is Involved in Cytokinin-Mediated Inhibition of Phosphate Transport and Phosphate Signaling in Rice (Oryza sativa L.)
Source: PLoS One. 2014 Nov 11;9(11):e112906. doi: 10.1371/journal.pone.0112906 (PMC4227850; doi:10.1371/journal.pone.0112906)
Supplement: Table S4 — Primer sequences for purple acid phosphatase genes. (DOCX) [file pone.0112906.s008.docx]

| **Table S4** Primer sequences for purple acid phosphatase genes | |
| --- | --- |
| OsPAP9b RT U: | ACCTACGATAACAGCAACTACGC |
| OsPAP9b RT L: | CATATCAGTTTTCGTCGCATGTA |
| OsPAP10a RT U: | GAGATAGATTTTGCCCCAGAACT |
| OsPAP10a RT L: | AGCTTCAAGCCACTTGTACTGA |
| OsPAP10c RT U: | TCTGGTACTCCGTCAAGATCG |
| OsPAP10c RT L: | CCCTCCATGTAGTGGTAGTT |
| OsPAP20b RT U: | GTCGGGGTCCAACCTCTACTA |
| OsPAP20b RT L: | CGCCCTGTTGCTGTTGTA |
| OsPAP23 RT U: | ATGGCATCCTAGAGGTGGTTAAT |
| OsPAP23 RT L: | CCACTGTTGCTCACTAGAGAAG |
| OsPAP27a RT U: | GCCACGTCCACAACTACGAG |
| OsPAP27a RT L: | CGTAGAGCAGCGACGTGTA |
